# Genomic markers analysis associated with resistance to *Alternaria alternata* (fr.) keissler—tomato pathotype, *Solanum lycopersicum* L.

**DOI:** 10.1270/jsbbs.22003

**Published:** 2022-08-26

**Authors:** Giti Alizadeh-Moghaddam, Mehdi Nasr-Esfahani, Zahra Rezayatmand, Mahdi Khozaei

**Affiliations:** 1 Department of Biology, Falavarjan Branch, Islamic Azad University, Isfahan, 84517-31167, Iran; 2 Department of Plant Protection Research, Isfahan Agricultural and Natural Resources Research and Education Center, AREEO, Isfahan, Iran; 3 Plant Biotechnology, Department of Biology, University of Isfahan, Isfahan, Iran

**Keywords:** early blight, genetic structure, ISSR markers, phenotypic profiles

## Abstract

*Alternaria alternata*, the causal pathogen of early blight (EB) disease, is one of the most important diseases in tomato, and other solanaceae family. We analyzed 35 tomato genotypes for quantitative/qualitative traits and biomass growth parameters, as well as the extent and structure of genetic variation associated with EB resistance. Phenotypic comparisons displayed significant differences in leaf blade width (24.95%), stem thickness (30.28%), foliage density (18.88%), and plant size (18.89%), with significant positive correlations with EB resistance (0.18–0.75). Correlation analysis showed that mature fruit size, thickness of fruit pericarp, and leaf type were significantly and negatively correlated with EB resistance (up to –0.41). The susceptible tomato seedlings represented significant reductions in biomass parameters. According to ISSR analysis, the highest resolving power (≥0.79) and heterozygosity (≥0.24) values revealed the presence of high genetic variability among the tomato genotypes. Bayesian model-based STRUCTURE analysis assembled the genotypes into 4 (best ΔK = 4) genetic groups. Combined phenotypic and molecular markers proved to be significantly useful for genetic diversity assessment associated with EB disease resistance.

## Introduction

Tomato, *Solanum lycopersicum* L. (Family: Solanaceae) is the most important consumed crop with culinary purpose in every healthy diet all over the world (Mata-Nicolás *et al.* 2020). Fruit quality attributes of tomato including fruit size, shape, color and flavor have been a main objective of tomato breeding programs to develop modern varieties. While fruit characteristics of tomato are the most important factors for commercial modern varieties, disease resistance is far more fundamental to tomato cultivation (Bertin and Génard 2018, Henareh *et al.* 2016, Salim *et al.* 2020).

In nature, tomato cultivation is exposed to a variety of pathogens affecting both yield and quality (Akhtar *et al.* 2019). Early blight (EB) disease, caused by *Alternaria alternata* (fr.) keissler and *A. solani* Ellis & G. Martin. (Family: Pleosporaceae), has emerged as the most destructive pre- and post-harvest disease of field and greenhouse tomato crops (Nasr-Esfahani *et al.* 2017a, Mulugeta *et al.* 2020). While fungicide applications are commonly used to control and reduce the damages, cultivation of disease-resistant genotypes is the most effective and sustainable method for integrated disease management (Nasr-Esfahani 2018a, Moghaddam *et al.* 2019). Therefore, identifying EB disease resistant genotypes and improving the genetic resistance of tomato to *A. alternata* (*AA*) are an effective approach toward development of tomato EB resistance (Nasr-Esfahani 2018b, Moghaddam *et al.* 2020).

Tomato genotypes display morphological variability in traits of leaves, flowers, and fruits. Qualitative and quantitative characteristics determine the agronomic value and taxonomic classification of plants. Variations in these characteristics are critical for varietal identification and selection, and breeders need suitable knowledge of tomato genetic diversity to identify and improve tomato genotypes with desirable characteristics in terms of agricultural traits, disease resistance, harvest yield and quality (Fernandes *et al.* 2018, Rosenqvist *et al.* 2019). Moreover, the development of phenotypic-genetic markers that are closely linked to EB disease resistance facilitates the introgression of phenotypic traits into marker-assisted selection in resistance breeding and identification of resistant tomato genotypes (Mongiano *et al.* 2020, Nafisa *et al.* 2020). Therefore, the morphological and genetic characterization of tomatoes is needed to identify and conserve EB-resistance sources (Mata-Nicolás *et al.* 2020). To date, a few literatures are available on the prognostic markers of EB disease resistance in tomato. Furthermore, there is no report about the association of phenotypic characteristics with molecular markers on EB disease resistance level in tomato. Moreover, to the best of our knowledge, usefulness of combined phenotypic and molecular characterization with EB disease resistance level hasn’t been investigated in tomato and there is no convincing evidence about whether phenotypic and genetic markers are linked to EB disease resistance. These approaches may make it easier to obtain the genetic determinants of early blight resistance and resistance QTL with candidate resistance genes in commercially adapted tomatoes (Anderson *et al.* 2021, Ashrafi and Foolad 2015).

The major objectives of this study were to: (i) assess phenotypic-genetic diversity, population structure, and relationships among tomato genotypes based on phenotypic and molecular markers, (ii) compare and combine phenotypic and genetic markers to identify genetic diversity of tomato genotypes to EB resistance. The degree of association between phenotypic and molecular markers would facilitate the development of resistant lines in a tomato breeding program.

## Materials and Methods

### Plant materials and plant cultural conditions

A collection of thirty five promising tomato commercial genotypes of exotic and domestic origin, and inbred line seeds (*Solanum lycopersicum* L.) described by Moghaddam *et al.* (2020), was used in this study, to characterize at both phenotypic and genetic levels (Supplemental Table 1).

An active *A. alternata*—tomato pathotype, isolate under KY322589 was received from Plant Protection Research Division, Isfahan Center for Research and Education in Agricultural Science and Natural Resources (AREEO), Isfahan, Iran (Nasr-Esfahani 2018a). The isolate was cultured on PDA at 24 ± 1°C for 15 days. To induce sporulation, cultures were incubated for 5 days on PDA under cool-white fluorescent light with 12 h/day light. Conidia were washed with sterile water and the spore suspension was adjusted to a concentration of 10^5^ spores mL^–1^. Pathogenicity tests were conducted in a greenhouse using *AA* spore suspension. The experiment was carried out in a completely randomized design with three replications and ten pots of each genotype per replication (35 × 10 × 3 = 1050 pots). Potted genotypes were inoculated in a greenhouse by spraying 3 times on the foliage at 3 day intervals and two separate growth stages, transplanting (6-week) and maturing (12-week after transplanting stage). Inoculated genotypes were covered by dark and clean plastic bags to increase humidity and infection, and then grown at 27 ± 1°C and 16-h photoperiod in the greenhouse. Disease severity was recorded 20 days after inoculation using the scoring scale of: 0, 5, 10, 25, 50, 75, or 100% at both stages (Moghaddam *et al.* 2020). The formulae in calculating the percent disease severity (PDS), in each replicate, was: PDS = (Σ RT × 100)/(S × N), where T is the total number of leaves in each category, R is the disease severity scale, N is the total number of leaves tested and S is the highest number in the scale (Nasr-Esfahani *et al.* 2017a, 2020, Naderi *et al.* 2020). Based on EB disease severity, the collection was previously screened into five distinct groups (Moghaddam *et al.* 2020); resistant (RR), partially resistant (PR)/susceptible (PS), susceptible (SS), and highly susceptible (HS) (Supplemental Table 1).

In this study, thirty five tomato genotypes tomato plants were hand-planted on July (2019) and on June (2020) in Agricultural and Natural Resources Research and Education Center of Esfahan-Iran, Latitude: 32° 39ʹ 8.86ʺ N and Longitude: 51° 40ʹ 28.63ʺ E. Healthy tomato seeds were surface sterilized by soaking in 2% sodium hypochlorite for 15 min and washed with distilled water three times. They were sown on seedling trays filled containing mix of sand-peat moss (in equal parts) steam-sterilized at 121°C for 30 min three times. The seedlings were grown under greenhouse conditions (27 ± 1°C, photoperiod 16 h and 65% relative humidity). After four weeks, two seedling per each genotype were transplanted into plastic pots (30 cm diameter) and filled with the same sterile soil, sand and peat moss. They watered twice a day. Plants were maintained at 27 ± 1°C in the greenhouse for two months (Bagheri *et al.* 2020, Nasr-Esfahani *et al.* 2017b). All tomato genotypes were grown according to a completely randomized experimental design with five replicates and ten plants *per* replicate.

### Morphological assessment and data collection

Morphological data were recorded during different phenological growth stages, pre-harvest morphological characterization and post-harvest fruit evaluation, based on ten randomly selected plants from each of the five replications, without disease and insect damage. They were selected as the average for subsequent measures for study use. Inflorescence data were collected on the 3rd fruit of the 3rd truss at the full maturity stage. Phenotyping was mainly based on 39 conventional descriptors: vegetative and reproductive traits correlated with EB disease, such as plant architecture (12), inflorescence (10), and fruit (17), on the basis of “Descriptors for Tomato (*Lycopersicon* spp.)” (IPGRI 1996, Salim *et al.* 2020) (Tables 1, 2). Among these evaluated morphological descriptors, 23 traits were qualitatively assessed whereas 16 traits were quantitatively measured. The number of leaves under 1st inflorescence, stem internodes length, vine length, width, length of leaf blade, stem thickness, plant size, stem pubescence density, foliage density, leaf type, anthocyanin coloration of leaf vein, and degree of leaf dissection were included to assess as architectural vegetative traits. The average of which was calculated to be used in this study. The width and length of petal, the width and length of sepal, stamen length, inflorescence type, corolla color, corolla blossom type, flower sterility type, style position, style shape, and style hairiness were recorded as reproductive performances and function-valued traits (Bannert *et al.* 2008). Characterization of fruit morphology; pedicel length, exterior color of immature fruit, predominant immature fruit shape, mature fruit shape, fruit shoulder shape, and intensity of exterior color were recorded as a measure of plant’s female success. Productivity and fruit yield of the tomato genotypes; mature fruit size, width and length of fruit, pericarp thickness, fruit pubescence, color and shape of seeds, and easiness of fruit to detach from the pedicel were recorded as a yield potential of all assessed genotypes.

### Biomass assessment

Biomass growth parameters (BGPs) including root fresh weight (RFW), root dry weight (RDW), stem diameter (SD), stem length (SL), stem fresh weight (SFW), stem dry weight (SDW), root diameter (RD), root length (RL), root volume (RV), and leaf length (LL) were recorded on three plants per genotype using digital scales, rulers and digital calipers. BGPs were measured two weeks after inoculation by a gentle up-rooting the tomato plants with no injury. Root volume was measured using the method of changes in the water volume (mm^3^). The main root from the point, where the first secondary root is initiated, and collar diameter (mm) was measured by using a digital caliper with accuracy of 0.01 mm. In addition, stem, root and the sixth leaf length from every tomato plants were expressed in cm. Dry-weight BGPs was taken place in an oven to evaporate the water at 80°C until all the water was evaporated, at the time when the weight of the sample no longer changes. The mean square of variances and mean comparison of the individual effect of inoculation treatment for the resistant and susceptible genotypes were evaluated (Álvarez-Gómez *et al.* 2021, Bagheri *et al.* 2020, Hashemi *et al.* 2020) (Table 3).

### ISSR parameters and data recording

In our previous study, total DNA was isolated from about 1 g of young leaf tissues of 35 tomato genotypes using the modified Cetyl Trimethyl Ammonium Bromide (CTAB) method (Moghaddam *et al.* 2020). Additionally, evaluations made on the basis of ISSR markers were also performed. Based on ISSR data obtained in our previous experiment, out of eleven primers of ISSR, nine primers were more polymorphic (Moghaddam *et al.* 2020). Therefore, we estimated the potential of these nine markers for evaluation of genetic variability by calculating; observed number of alleles per locus (Na), effective number of alleles per locus (Ne) resolving power (Rp), expected heterozygosity (He), and observed heterozygosity (Ho) (Tehrani *et al.* 2020) (Supplemental Table 3).

### Statistical analysis

Normality test for the morphological variables, combined variance analysis, and comparison means by LSD test were performed using SAS v.9.4 (SAS 2004). Genotype Clustering based on the morphology characteristics was performed using the SAHN in NTSYSpc v.2.1. To illustrate multiple dimension distribution of tomato genotypes based on the morphology characteristics, principal component analysis (PCA) was also projected by NTSYSpc v.2.1 (Nasehi *et al.* 2019). In evaluating the correlation between morphological traits and EB resistance, ‎the relationship between trait changes and resistance level in all genotypes was compared, and the ‎correlation was calculated using Pearson correlation coefficient (Ghasemi *et al.* 2014, Hashemi *et al.* 2020). Genetic diversity indices were estimated by GenAlex v.6.5. The values of Na, Ne, He and Ho were determined using the protocol described by Yeh *et al.* (1997). The Rp values were calculated for each ISSR primer locus as Pr = ΣI_b_, where I_b_ or band informativeness is represented on a scale of 0–1 and is defined as I_b_ = 1 – (2 × |0.5 – p|), where p is the portion of the samples containing the observed band (Amiryousefi *et al.* 2018). Principal coordinate analysis (PCoA) was performed to visualize patterns of genetic diversity using GenAlex v.6.5 (Peakall and Smouse 2012).

To explore genetic structure of two data sets (genetic and phenotypic data) of 35 tomato genotypes, STRUCTURE 2.3.4 was applied allowing for admixture and correlated allele frequencies (Pritchard *et al.* 2000, 2010). Admixture models are more flexible than non-admixture models because of a common feature for real data. To obtain an accurate estimation of the best K (number of clusters) for each data set, a continuous series of Ks (1–10) in ten independent runs for each K was performed with a burn-in period of 50000 steps and a run length of 500000 Markov Chain Monte Carlo (MCMC) iterations. Further, ln P(D|K) (the logarithm of the probability of the data given K) was calculated by implementing the Evanno ΔK statistics (Evanno *et al.* 2005) using the program STRUCTURE HARVESTER (Earl and von Holdt 2012). Finally, a dendrogram was produced according to the Unweighted Pair Group Method with Arithmetic Average (UPGMA) using NTSYS-pc v.2.1 software to evaluate the relationships among the genotypes and more importantly, to classify the genotypes based on genetic-phenotypic markers and EB resistance level.

## Results

### Phenotypic variation and multivariate analysis

The 39 morphological traits analysis were carried out to quantify phenotypic differences among tomato genotypes with different levels of resistance to *A. alternata* (Tables 1, 2). Based on the mean disease severity of two growth stages, the tomato genotypes were classified into five classes in which less than 16% was considered as resistant (R), 17–27% as partially resistant (PR), 28–31% as partially susceptible (PS), 32–40% as susceptible (S) and 40% or more as highly susceptible genotypes (HS) to *AA* infection (Supplemental Table 1). Overall, considering both the parameters (genotype and growth stage) Esfahan Local, Rio Grande and Turkish Cherry were the most considerable genotypes with high resistance level. On the contrary, CH American, Ameera, Hedieh and Retinto were highly susceptible genotypes (Supplemental Table 1).

A total of 16 quantitative and 23 qualitative characters describing the variability of this collection for leaves, flowers, fruits and plant architecture were evaluated to estimate mean squares as well as correlation coefficients with EB disease resistance (Tables 1, 2, Supplemental Table 2). Characterized morphological descriptors displayed a highly significant variation in all 11 morphological quantitative traits (p < 0.001), indicating the existence of wide range variability collection (Table 1). The highest morphological variability was found in most quantitative traits related to fruit and flower characteristics: sepal width (93.31%), petal width (77.88%) under 1st inflorescence, thickness of fruit pericarp (73.61%), mature fruit size (38.85%) and fruit length (36.85). The lowest coefficient of variation was recorded for architecture characteristics: stem internode length (15.12%), petal length (17.82%) and leaf blade length (19.05%) (Table 1).

Further statistical analysis of the correlation between the quantitative traits and the level of EB disease resistance was reported in Table 1 and Supplemental Table 2. The correlation coefficient analysis with resistance to *AA* revealed significant associations with most quantitative traits, except for fruit width, petal length and stamen length. The highest significant positive correlation with EB disease resistance was obtained for 7 different quantitative characters related to most architecture characteristics: number of leaves under 1st inflorescence (r = 0.75**), vine length (r = 0.58**), leaf blade width (r = 0.41**), sepal width (r = 0.36**), stem thickness (r = 0.31**), leaf blade length (r = 0.21**), and stem internode length (r = 0.18**) (p < 0.001) (Table 1). The highest significant negative correlation with EB disease resistance was obtained for 4 quantitative characters related to most fruit characteristics: fruit length (r = –0.36**), mature fruit size (r = –0.34**) and thickness of fruit pericarp (r = –0.32**) (p < 0.001) (Table 1).

The results of variance analysis for the qualitative traits showed that they were significantly differed among the 35 tomato genotypes, except for degree of dissection, anthocyanin coloration of leaf vein, exterior color of immature fruit, fruit pubescence and intensity of exterior color (Table 2). The highest significant variation (p < 0.001) was recorded for the qualitative traits related to fruit and flower characteristics: exterior color of mature fruit (4.83), immature fruit shape (4.46%), mature fruit shape (3.66%), flower sterility type (2.63%), corolla color (2.03%), fruit shoulder shape (1.63%), and to some architecture characteristics: leaf type (3.40%) and foliage density (2.71%). The higher significant variation was recorded for plant size (2.60%), stem pubescence density (2.51%), seed shape (2.03%), and corolla blossom type (1.54%) (p < 0.05) (Table 2). Furthermore, the highest significant positive correlation with EB disease resistance was recorded for stem pubescence density (r = 0.31**), foliage density (r = 0.27**), plant size (r = 0.18**), flower sterility type (r = 0.64**) and fruit shoulder shape (r = 0.33**) (p < 0.001) (Table 2). Similarly, the higher significant positive correlation was recorded for fruit and flower characteristics: corolla blossom type (r = 0.18*), inflorescence type (r = 0.16*), and seed shape (r = 0.14*) (p < 0.05) (Table 2). The highest significant negative correlation with EB disease resistance was observed for the qualitative characters: leaf type (r = –0.41**) and mature fruit shape (r = –0.39**) (p < 0.001) (Table 2).

**Fig. 1.**
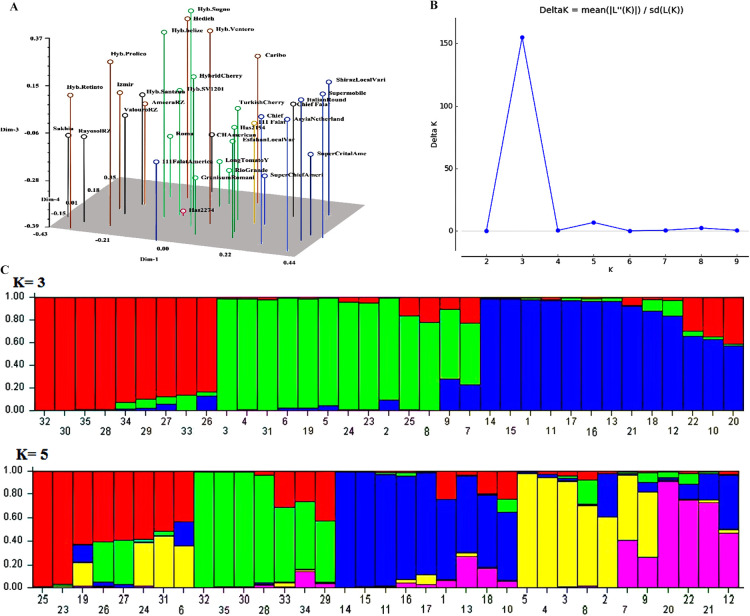
(A) Principal coordinate analysis (PCA) using phenotypic markers efficient similarities among 35 tomato genotypes. Each color represents a cluster, and each dot represents a genotypes; (B) Graph showing the best value of K at 3; (C) Admixture plot showing clustering of 35 tomato genotypes into three and five clusters based on the phenotypic data using Bayesian-based clustering analysis. A vertical number bar represents each genotype code. The colored sections in a bar show membership coefficients of the genotypes in the different clusters.

In the UPGMA analysis, all 39 traits of 35 accessions were used and grouped into 2 major groups (Supplemental Fig. 1). The Cophenetic correlation coefficient based on Jaccard’s similarity coefficient was 0.68%, indicating a reasonable diversity between the genotypes. Based on the reference line, the genotypes branched into six clades (I, II, III, IV, V, VI), which largely coincided with the EB disease resistance level. The lowest similarity coefficient (0.29–0.31), which is indicative of the highest morphological diversity, was observed in Esfahan Local as a resistant genotype (clade III). The fourth and sixth clades comprised the most of tomato genotypes with the highest susceptibility (CH American, Hedieh, Ameera, Izmir), with 40–60% similarities (Supplemental Fig. 1). PCA was performed to assess the displacement of the genotypes and to further confirm the clustering pattern obtained from the UPGMA. PCA showed a significant clustering of tomato genotypes that were plotted into six sub-plots, representing the significant phenotypic differences and differentiation among the genotypes (Fig. 1A).

### Biomass analysis

Analysis of variance of biomass growth parameters (BGPs) data in *AA* inoculated tomatoes (Table 3) showed that genotype and inoculation treatment factors had a significant effect on changes in BGP (p ≤ 0.01). However, the interaction of these two factors had no significant effect on BGP except SDW, RDW and SL (Table 3). Comparison of the mean individual effect of inoculation treatment on BGP traits showed significant reductive changes in BGPs in inoculated samples compared to the controls, non-inoculated genotypes. Individual effect of genotype factor also showed a significant difference between BGP traits and resistant and susceptible genotypes. Overall, the mean value of BGPs was significantly higher in non-inoculated resistant tomato genotypes compared with inoculated ones. The highest SFW and SDW were in the non-inoculated resistant genotype, Shiraz Local, with 4.33 and 2.52 (gr), and SFW and SDW in Esfahan Local 3.92 and 1.48 (gr), respectively. The highest SD, RD, LL, SL, RL and RV in the resistant genotype, Shiraz Local, with 7.67 (mm), 5.58 (mm), 8.11 (cm), 19.11 (cm), 12.03 (cm), and 4.57 (mm^3^), respectively. The results of BGP traits showed that the inoculated resistant and susceptible tomato seedlings with A. alternata had significant effects on all growth parameters, including the dry and fresh biomass compared to the controls (Table 3) (p ≤ 0.01).

### Genotyping and multivariate analysis

**Fig. 2.**
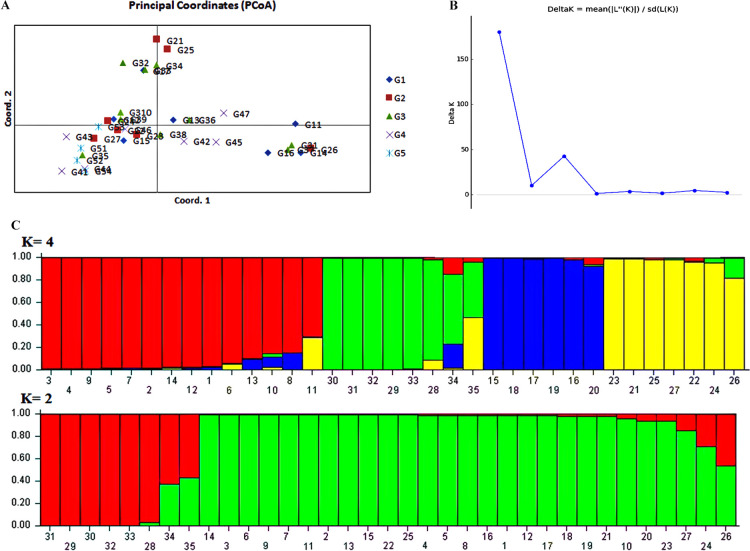
(A) Two-dimensional representations of the first two axes of the principal coordinates analysis (PCoA) from the matrix of genetic distances of 35 tomato genotypes (G1: highly susceptible genotypes, G2: susceptible genotypes, G3: partially susceptible genotypes, G4: partially resistant genotypes, G3: resistant genotypes; (B) Graph showing the best value of K at 2; (C) Admixture plot showing clustering of 35 tomato genotypes into two and four clusters based on the molecular data using Bayesian-based clustering analysis. A vertical number bar represents each genotype code. The colored sections in a bar show membership coefficients of the genotypes in the different clusters.

Mean values of Ne, Na, PIC, Rp, He and Ho were 1.35, 1.75, 0.28, 0.79, 0.24 and 0.21, respectively (Supplemental Table 3). The Rp values varied between 0.31 for INC3 and 1.47 for 430 followed by UBC811 (Rp = 1.25). Genetic diversity is assessed as the amount of actual or potential heterozygosity. Expected heterozygosity was higher than observed heterozygosity at all the loci, except to UBC848 and UBC840. The observed and expected heterozygosity values ranged from 0.03 (430) to 0.41 (UBC840) and from 0.08 (430) to 0.34 (UBC840), respectively (Supplemental Table 3). The results obtained from nine informative primers were subjected to conduct cluster analysis (Supplemental Fig. 2). The similarity was constructed using the Jaccard coefficient method. The 35 tomato genotypes were grouped into two major clusters and five sub-groups (I, II, III, IV, and V) at a coefficient of 0.46. The similarity coefficient value ranged from 0.29 to 0.98. The lowest similarity coefficient (0.29–0.47), indicating the highest genetic diversity, was observed in Italian Round (clade I) followed by clades IV and V, which contained Roma and H.a.s. 2274 as the partially resistant genotypes, respectively. Clade III consisted of Esfahan Local, Turkish Cherry and Rio Grande as the resistant genotypes, followed by clade IV which contained the most highly susceptible genotypes; Hedieh, Ameera, Izmir, Super Cristal, Sogno, Retinto. Furthermore, the partially resistant and susceptible genotypes did not display such a clear distinction and were distributed all over the clades (Supplemental Fig. 2). Principal coordinates analysis (PCoA) was performed to provide spatial representation of the genetic diversity among the 35 tomato genotypes (Fig. 2A). The first two principal coordinates accounted for 28.81% of the total variance (17.75% and 11.06%, respectively).

### Genetic and phenotypic structure analysis

To study the phenotypic and genetic structure of tomato genotypes and the genetic relationship among samples, two distinct Bayesian assignment analyses were performed. The results of Bayesian analysis of phenotypic structure indicated that the most likely number of subgroups and ΔK method in the tomato diversity panel was K = 3 followed by K = 5 indicating that the genotypes could be grouped into three major clusters and five sub-clusters (Fig. 1B). At K3, the three major groups comprised (1) the most highly susceptible and partially susceptible genotypes (n = 4), (2) the most highly susceptible, partially susceptible and susceptible genotypes (n = 7), (3) the most resistant and partially resistant genotypes (n = 8), and 16 genotypes including the most partially resistant and susceptible genotypes (21.5%) showed mixture position (Fig. 1C). At K5 the five major groups encompassed (1) the highly susceptible and partially susceptible genotypes (n = 4), (2) the highly susceptible and partially susceptible genotypes (n = 2), (3) the susceptible and partially susceptible genotypes (n = 4), (4) the resistant and partially resistant genotypes (n = 3), and (5) the partially susceptible genotype (Granisum Romani) (n = 1), and 21 genotypes including the most partially resistant and susceptible genotypes (60%) showed mixture position (Fig. 1C). The phenotypic analyses (UPGMA and PCA) (Supplemental Fig. 1 and Fig. 1A, respectively) indicated a similar result which is consistent with the STRUCTURE results at K = 3 (Fig. 1C).

Based on genetic data, the maximum log-likelihood given by STRUCTURE and ΔK method was K = 2 followed by K = 4 indicating that the 35 tomato genotypes could be grouped into two main populations and four subpopulations (Fig. 2B). At K2, the two main groups comprised (1) the most highly susceptible, four partially susceptible and susceptible genotypes (n = 6), (2) 21 genotypes assigned into second groups, the most partially resistant and susceptible genotypes, and 8 genotypes (7%) showed mixture position (Fig. 2C). At K4 the four major groups encompassed (1) the most highly susceptible, susceptible and partially susceptible genotypes (n = 6), (2) the most partially susceptible and susceptible genotypes (n = 6), (3) the most highly susceptible and four partially susceptible (n = 6), and (4) the all resistant and partially resistant genotypes (n = 10). Accordingly, out of 35 genotypes, 7 genotypes including the most partially resistant and susceptible genotypes showed mixture position (5%) (Fig. 2C). The highly susceptible genotypes formed one cluster at K = 2 and at K = 4. Whereas the all resistant genotypes were more similar and formed one genetic group at K = 4. The genetic analyses (UPGMA and PCoA) (Supplemental Fig. 2 and Fig. 2A, respectively) indicated a similar result which is consistent with the STRUCTURE results at K = 4 (Fig. 2C).

### Combined analysis of morphological and molecular markers

**Fig. 3.**
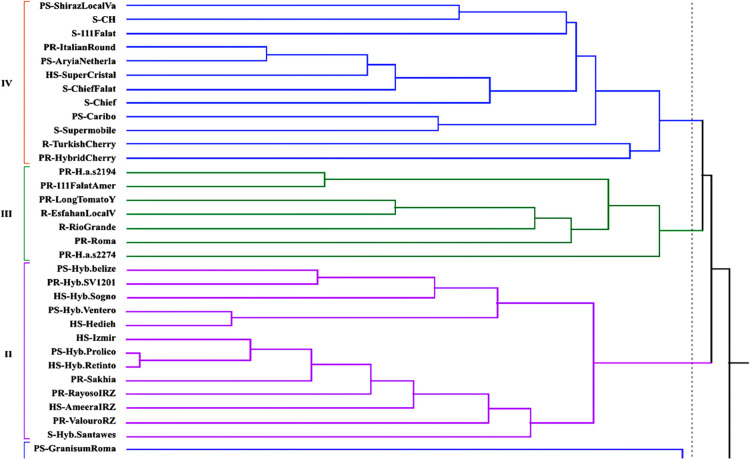
Clustering analysis based on morphological, molecular and resistance levels data of 35 tomato genotypes using Jaccard’s similarity coefficient and UPGMA. HS (Highly susceptible), S (Susceptible), PS (Partially susceptible), PR (Partially resistant), R (Resistant).

The grouping pattern of combined phenotypic and genetic data based on EB disease resistance using UPGMA method showed two major clusters and four clades at a coefficient of 0.36 (Fig. 3). The similarity was constructed using Jaccard coefficient method (0.74), and the similarity coefficient value ranged from 0.34 to 0.87. The second and fourth clades (II, IV) were the biggest with 25 genotypes comprising of the most susceptible, partially susceptible, and highly susceptible genotypes. The third clade (III) contained 7 resistant ant partially resistant genotypes. The first clade (I) was the smallest group containing 3 highly and partially susceptible genotypes. The lowest similarity coefficient (0.37–0.39) was observed in Granisum, Romania, H.a.s. 2274, Turkish Cherry and Hybrid Cherry genotypes, which had the longest branch that showed a great differentiation with other genotypes (Fig. 3).

## Discussion

The recent progresses presented a step-change towards diversity analysis in plant phenotyping and breeding programs using molecular and morphological markers with high potential (Nankar *et al.* 2020, Rosenqvist *et al.* 2019, Tripodi *et al.* 2018). The top breeding priority in tomato has been yield, productivity, and disease resistance to maintain the sustainable production in challenging environment (Bhattarai *et al.* 2018, Singh *et al.* 2017). The identification of EB disease resistant genotypes with acceptable horticultural traits is an emerging approach in selection intensity and selection accuracy (Awan *et al.* 2018, Chaudhari *et al.* 2019, Nasr-Esfahani 2020). Phenotypic and genotypic data can be used to estimate relationships between individual genotypes with the purpose of optimizing characterization and determining of functional alleles underlying trait variation (Sacco *et al.* 2015, van Eeuwijk *et al.* 2019). Therefore, it is necessary to determine and classify the valuable quantitative and qualitative traits for genetic improvement of biological population (Yadeta-Dabalo *et al.* 2020).

In the present study, significant variations of 39 morphological characteristics were documented among different tomato genotypes. These genotypes were selected to represent a range of phenotypic diversity in relationship to EB disease resistance. As the evaluation was only performed under controlled environment conditions, our interest was directed on genotypic effects. The analysis of variance revealed the presence of a high variability among the tested genotypes in all quantitative traits. The characteristics such as, sepal width, petal width, thickness of fruit pericarp, mature fruit size manifested a wide range of variability. Previously, flower traits are considered as key traits involved in evaluation of tomato local forms and breeding lines (Figàs *et al.* 2018, Nankar *et al.* 2020). Salim *et al.* (2020) analyzed 22 tomato inbred lines and found a similar level of variability for length, diameter and pericarp thickness of fruit. Current results demonstrated that the thickness of fruit pericarp and mature fruit size would be useful for genotype distinctness analysis (Fiorani and Schurr 2013, Salim *et al.* 2020). Moreover, fruit morphology is considered challenging and time-consuming because of the quantitative nature of the characteristics (Fiorani and Schurr 2013). The levels of variation observed between tomato genotypes for architecture characteristics: internode length, petal length, leaf blade length, stamen length, and leaf blade width were also significant. Architecture characteristics such as size of leaves, height of internode length and stem diameter have been reported as part of diagnostic indices (Chitwood *et al.* 2013, Yamamoto *et al.* 2017). By characterizing 971 T-DNA lines, Jáquez-Gutiérrez *et al.* (2019) showed that the genetic dissection of tomato leaf architecture was regulated by homologous gene modules.

In this study, the characterization of qualitative characteristics revealed that a high diversity existed among these promising genotypes, which can be utilized to differentiate genotypes. Our findings showed that the level of variance of mature fruit shape, fruit shoulder shape, foliage density, plant size, stem pubescence density, was highly significant. In agreement with our results, previous studies (Bhattarai *et al.* 2018, Figàs *et al.* 2018, Salim *et al.* 2020) found a wide variation in fruit and flower shape. Likewise, it has been declared that four genes controlled the number of locule and tomato fruit shape, elongated and flat (Bhattarai *et al.* 2018, Prieto *et al.* 2018). Notably, statistical analysis was used to detect the significance of the association between the phenotypic traits and EB resistance pattern in the tomato genotypes. The results demonstrated a significant positive correlation between stem pubescence density, foliage density, plant size, and fruit shoulder shape with EB disease resistance. These phenotypic characteristics as well as EB disease severity showed that they were more pronounced in plant maturation rather than early growth stage (Moghaddam *et al.* 2019, 2020). Mature and immature fruit shape, exterior color of mature fruit, and leaf type characteristics displayed significant negative correlation with EB disease resistance level. Moreover, it was suggested that leaf type and fruit shape were valuable diagnostic traits, which can be used to differentiate the tomato genotypes (Chitwood *et al.* 2013, Salim *et al.* 2020). Besides of morphological traits, significant reductions in biomass growth parameters, together with length of root and stem, and root volume parameters were observed on susceptible tomato seedlings, which in turn represented key traits crucial for tomato’s functionality. Both the fresh and dry stem and root weights of tomato genotypes were affected significantly, although at a different degree and in relation to EB disease.

In the present study, the analysis of ISSR data with the mean values of Ne (1.75), Rp (0.79), PIC (0.28) and He (0.24) showed a considerable level of genetic divergence among the examined genotypes. Considering that Rp and He mean values were affirmed to assess polymorphism within genotypes depended on allele frequency distributions (Albrecht *et al.* 2010). Our results indicated that there was enough genetic diversity among the studied genotypes to support the presence of different disease resistance levels. According to that observed in previous studies, *S. lycopersicon* had an expected heterozygosity mean value (He = 0.25) lower than that of other tomato species such as *S. lycopersicum* var. *cerasiforme* (He = 0.38) and *S. pimpinellifolium* (He = 0.58) (Ranc *et al.* 2008). Therefore, our results suggested the detection power of tested markers for variations or polymorphisms.

Our population structure analysis identified two main groups and four subgroups, which it also agreed with the UPGMA analyses of the genetic and phenotypic data. Accordingly, the clustering pattern of these data consistently explained a similar genetic structure among the highly susceptible genotypes (Figs. 1C, 2C). Furthermore, the results of clustering analysis displayed a genetic admixture between the studied genotypes. In both the structure patterns, the highly susceptible genotypes, Izmir, Ameera and Retinto, and resistant genotypes, Rio Grande and Turkish Cherry, were grouped in two distinct groups. The partially susceptible and susceptible tomato genotypes have been observed to show a great genetic diversity and were distributed all over the clusters. The high agreement of the UPGMA with the population structure is corroborated by the findings for other crops such as safflower (Ambreen *et al.* 2018), purple yam (Agre *et al.* 2019), and Taramira genotypes (Zafar-Pashanezhad *et al.* 2019). In order to address whether the molecular analysis produced a similar diversity pattern with the phenotypic architecture, and to examine genetic-phenotypic correlation with EB disease resistance level, a combined analysis was carried out using all the phenotypic data, molecular data, and EB disease resistance data across the 35 tomato genotypes. The cluster analysis showed that most of the resistant genotypes were grouped in a distinct cluster (Fig. 3, clade III) accompanied with the partially resistant genotypes from the highly susceptible ones. The highest distance obtained between the resistant and the susceptible genotypes can be compromised due to the high genetic diversity within these evaluated genotypes. The diagnostic potential of phenotypic data coupled with ISSR data for EB disease resistance in this study is corroborated by previous studies (Hashemi *et al.* 2019, Vargas *et al.* 2020). Recently, Mahmoud and El-Fatah (2020) showed that sixty *Faba bean* genotypes obviously were clustered into three major groups using molecular and biochemical markers according to their resistance to *Fusarium* wilt. Clearly, our clustering analysis displayed notable genetic diversity in terms of morphological traits to discriminate resistant from susceptible tomato genotypes.

To our knowledge, the present study is the first attempt to understand the extent of agreement/correlation between genetic and phenotypic markers associated with EB disease resistance in tomato plants. Our study revealed feasibility of phenotypic and genetic markers to identify sources of EB disease resistance. Based on morphological data, the highest significant negative correlation with EB disease resistance was observed for leaf type, mature fruit size, thickness of fruit pericarp, sepal length, petal width, and fruit shape characteristics. These tomato characteristics can be considered as favorable attributes for genetic improvement strategies through quantitative and biometrical genetics. According to ISSR analysis, the degree of genetic differentiation among tomato genotypes was high and significant for most resolving power and heterozygosity values. These morphological and molecular markers assembled the genotypes into 4 (best ΔK = 4) genetic groups. As a result of clustering analysis, the tomato genotypes were grouped in accordance with their EB resistance level. As a further line of research, these findings could be used as potential patterns in morphological trait variations and genetic diversity to identify diverse tomato genotypes with strong EB resistance.

## Author Contribution Statement

GAM, ZR and MNE designed the study. GAM and MNE provided support for conducting the research, developed the plant materials, and wrote the manuscript. GAM analyzed results, and wrote the paper. MK provided methodology and revised the manuscript. MNE and ZR supervised the study, and validated the results. All authors have read and agreed to the published version of the manuscript.

## Supplementary Material

Supplemental Figures

Supplemental Tables

## Figures and Tables

**Table 1.** Mean squares and correlation coefficient for sixteen quantitative characters of 35 tomato genotypes to *A. Alternata*

S.O.V	DF	Mean Square								
		Vine length	Stem internode length	Number of leaves under 1st inflorescence	Stem thickness	Leaf blade width	Leaf blade length	Mature fruit size	Fruit width	Fruit length
Genotype	34	2272.795**	6.165**	186.448**	30.855**	7.096**	13.575**	20.200**	19.933**	17.875**
Error	140	27.974	0.259	3.909	0.422	0.219	0.467	0.001	0.001	0.004
CV (%)		26.16	15.12	32.55	30.28	24.95	19.05	38.85	35.83	36.85
Correlation coefficient with resistance to *A. Alternata*		0.580**	0.181**	0.749**	0.306**	0.405**	0.212**	–0.341**	–0.060^ns^	–0.362**
S.O.V	DF	Pedicel length	Thickness of pericarp	Petal length	Petal width	Sepal length	Sepal width	Stamen length		
Genotype	34	2.666**	18.294**	0.257**	0.646**	0.254**	2.041**	0.156**
Error	140	0.000	0.000	0.004	0.112	0.002	0.002	0.002
CV (%)		26.53	73.61	17.82	77.88	26.45	93.31	20.02
Correlation coefficient with resistance to *A. Alternata*		–0.174*	–0.319**	0.117^ns^	–0.174*	–0.300**	0.356**	0.096^ns^

Note: ns: not significant, *: significant at p < 0.05, **: significant at p < 0.01.

**Table 2.** Mean squares and correlation coefficient of twenty three qualitative characters of 35 tomato genotypes to *A. Alternata*

S.O.V	DF	Mean	Std. deviation	CV (%)	Minimum	Maximum	Correlation coefficient with resistance to *A. Alternata*
Plant size	34	2.6000*	0.49130	18.89	2.00	3.00	0.180**
Stem pubescence density	34	2.5143*	0.50123	19.93	2.00	3.00	0.308**
Foliage density	34	2.7143**	0.51257	18.88	1.00	3.00	0.266**
Leaf type	34	3.4000**	0.83735	24.63	2.00	5.00	–0.405**
Degree of leaf dissection	34	2.8000^ns^	0.46732	16.69	1.00	3.00	0.063^ns^
Anthocyanin coloration of leaf vein	34	1.1714^ns^	0.37796	32.26	1.00	2.00	–0.033^ns^
Exterior color of immature fruit	34	1.8857^ns^	0.78680	41.72	1.00	5.00	–0.115^ns^
Fruit pubescence	34	1.3143^ns^	0.57593	43.82	1.00	3.00	–0.090^ns^
Predominant immature fruit shape	34	4.4571**	2.25326	50.55	3.00	8.00	–0.308**
Exterior color of mature fruit	34	4.8286**	0.56149	11.63	3.00	5.00	–0.344**
Intensity of exterior color	34	2.1429^ns^	0.64071	29.89	1.00	3.00	–0.108^ns^
Mature fruit shape	34	3.6571**	2.07535	56.75	1.00	9.00	–0.393**
Easiness of fruit to detach from the pedicel	34	2.0857^ns^	0.80841	38.76	1.00	3.00	0.226**
Fruit shoulder shape	34	1.6286**	0.72261	44.37	1.00	3.00	0.325**
Seed shape	34	2.0286*	0.65591	32.33	1.00	3.00	0.144*
Seed color	34	1.4571^ns^	0.49959	34.28	1.00	2.00	–0.180**
Inflorescence type	34	1.4286^ns^	0.60104	42.07	1.00	3.00	0.161*
Corolla color	34	2.0286**	0.16708	8.23	2.00	3.00	0.309**
Corolla blossom type	34	1.5429*	0.49959	32.38	1.00	2.00	0.175*
Flower sterility type	34	2.6286**	0.48457	18.43	2.00	3.00	0.638**
Style position	34	1.8276**	0.61215	33.49	1.00	4.00	–0.264**
Style shape	34	1.1143^ns^	0.46556	41.78	1.00	3.00	–0.059^ns^
Style hairiness	34	1.1143**	0.31907	28.63	1.00	2.00	0.266**

Note: ns: not significant, *: significant at p < 0.05, **: significant at p < 0.01.

**Table 3.** Mean squares of variance analysis and mean comparison of the individual effect of inoculation treatment for the evaluated biomass parameters, for susceptible and resistant tomato genotypes to early blight disease, *A. alternata*

S.O.V		DF	Mean Square									
			Stem fresh weight (gr)	Stem dry weight (gr)	Root fresh weight (gr)	Root dry weight (gr)	Stem diameter (mm)	Root diameter (mm)	Leaf length (cm)	Stem length (cm)	Root length (cm)	Root volume (m^3^)
Inoculation treatment (A)		1	5.19**	0.066**	0.471**	0.0016^ns^	1.417*	0.401^ns^	10.77**	0.41^ns^	19.96**	8.95**
Genotype (B)		5	6.11**	0.171**	0.081^ns^	0.0024**	0.315^ns^	2.143^ns^	4.52**	19.21**	10.03^ns^	2.66**
A * B		5	0.76^ns^	0.026*	0.121^ns^	0.0023**	0.604^ns^	0.842^ns^	0.75^ns^	2.61^ns^	7.99^ns^	1.40*
Error		55	0.43	0.016	0.161	0.0011	0.421	0.746	0.71	6.17	6.04	0.45
CV (%)			24.1	23.3	25	15.1	18.01	20.8	16.5	14.64	22.1	26.6
Inoculation treatment			Stem fresh weight (gr)	Stem dry weight (gr)	Root fresh weight (gr)	Root dry weight (gr)	Stem diameter (mm)	Root diameter (mm)	Leaf length (cm)	Stem length (cm)	Root length (cm)	Root volume (mm^3^)
Non-infected			4.968 ^a^	0.469 ^a^	2.107 ^a^	0.702 ^a^	4.46 ^a^	5.01 ^a^	7.59 ^a^	17.68 ^a^	11.95 ^a^	3.03 ^a^
Infected			3.225 ^b^	0.398 ^b^	1.883 ^b^	0.711 ^a^	4.12 ^a^	4.33 ^b^	5.81 ^b^	17.20 ^a^	10.53 ^b^	2.4 ^b^
Statistical level			1%	1%	1%	1%	5%	1%	1%	1%	1%	1%
Genotype group			Stem fresh weight (gr)	Stem dry weight (gr)	Root fresh weight (gr)	Root dry weight (gr)	Stem diameter (mm)	Root diameter (mm)	Leaf length (cm)	Stem length (cm)	Root length (cm)	Root volume (mm^3^)
Resistant			4.762 ^a^	0.5701 ^a^	2.903 ^a^	0.747 ^a^	4.552 ^a^	5.558 ^a^	7.64 ^a^	18.002 ^a^	10.48 ^a^	3.46 ^a^
Susceptible			3.474 ^b^	0.396 ^b^	1.509 ^b^	0.680 ^b^	3.899 ^b^	4.110 ^b^	7.04 ^b^	17.17 ^b^	9.079 ^b^	3.06 ^a^
Statistical level			1%	1%	5%	5%	5%	1%	1%	1%	5%	1%
Inoculation treatment × Genotypes (I × G)			Stem fresh weight (gr)	Stem dry weight (gr)	Root fresh weight (gr)	Root dry weight (gr)	Stem diameter (mm)	Root diameter (mm)	Leaf length (cm)	Stem length (cm)	Root length (cm)	Root volume (mm^3^)
Non-infected	Shiraz Local		4.333 a	2.523 a	2.387 ab	1.018 cd	7.671 ab	5.581 ab	8.11 a	19.111 a	12.03 ab	4.57 bcd
	Esfahan Local		3.922 ab	1.489 b	2.403 ab	1.118 a	7.678 a	5.589 a	7.87 ab	18.997 b	11.52 b	4.50 bcd
	2274 H.a.s		2.577 cde	0.919 cde	2.411 a	1.208 a	7.50 cd	5.588 a	7.03 b	19.106 ab	14.14 a	4.48 bcd
	Caribo		2.385 cde	0.799 e	2.375 b	1.024 cd	7.42 d	5.583 ab	6.73 bc	19.001 b	12.00 ab	3.31 d
	Hedieh		2.528 cde	0.875 de	2.391 ab	0.927 d	7.505 bcd	5.579 ab	6.47 c	19.102 ab	12.16 ab	4.40 cd
	Ameera		2.408 cde	0.851 e	2.415 a	1.033 cd	7.508 bcd	5.572 abc	8.03 a	17.997 d	11.85 b	4.16 cd
Infected	Shiraz Local		3.030 bcd	1.413 bc	2.088 b	0.915 d	7.663 abcd	5.577 ab	8.00 a	19.095 abc	12.72 ab	4.60 ab
	Esfahan Local		3.132 bc	1.407 bcd	2.303 a	1.008 d	7.54 cd	5.585 a	6.52 c	17.992 d	10.54 bc	4.33 bcd
	2274 H.a.s		2.538 cde	0.878 de	2.261 ab	1.021 cd	7.501 bcd	5.574 b	7.13 b	17.989 de	13.03 ab	5.16 a
	Caribo		2.193 cde	0.896 cde	2.116 b	0.707 e	7.397 d	5.571 b	6.36 bc	17.983 e	11.74 b	3.10 d
	Hedieh		2.162 de	0.772 e	2.231 ab	0.521 ef	7.486 bcd	5.566 abc	6.57 bc	17.987 e	11.39 ab	4.16 cd
	Ameera		2.003 e	0.778 e	2.243 ab	0.880 de	7.492 bcd	5.557 c	7.79 ab	17.981 ef	10.01 bcd	3.51 cd
Statistical level			1%	1%	5%	1%	5%	1%	1%	1%	1%	1%

Note: the heights and diameters: centimeter (cm), weights: grams (gr), volumes: millimeters (mm^3^). ns: not significant, *: significant at p < 0.05, **: significant at p < 0.01. DF: degree of freedom, CV: coefficient of variation. Means in each column having same letter are not significantly different according to LSD test.
