# Supplementary material for: Genomic markers analysis associated with resistance to Alternaria alternata (fr.) keissler—tomato pathotype, Solanum lycopersicum L
Source: Breed Sci. 2022 Aug 26;72(4):285–96. doi: 10.1270/jsbbs.22003 (PMC9868332; doi:10.1270/jsbbs.22003)
Supplement: Supplementary file 1 — Supplemental Figures [file 72_285_s1.pdf]

doi: 10.1270/jsbbs.22003

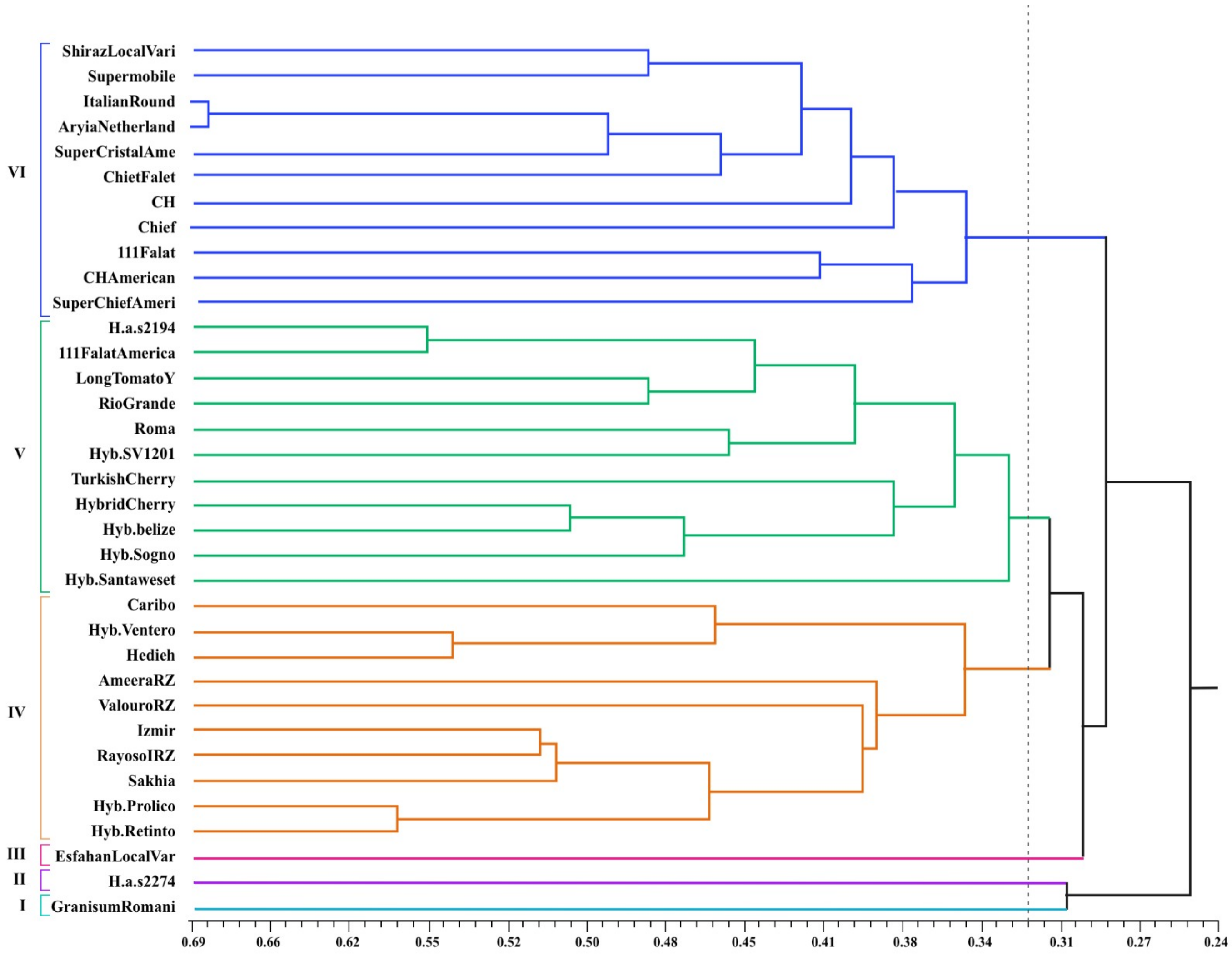

Supplemental Fig. 1. Clustering analysis based on 39 morphological attributes of 35 tomato genotypes using Jaccard's similarity coefficient and UPGMA algorithm.

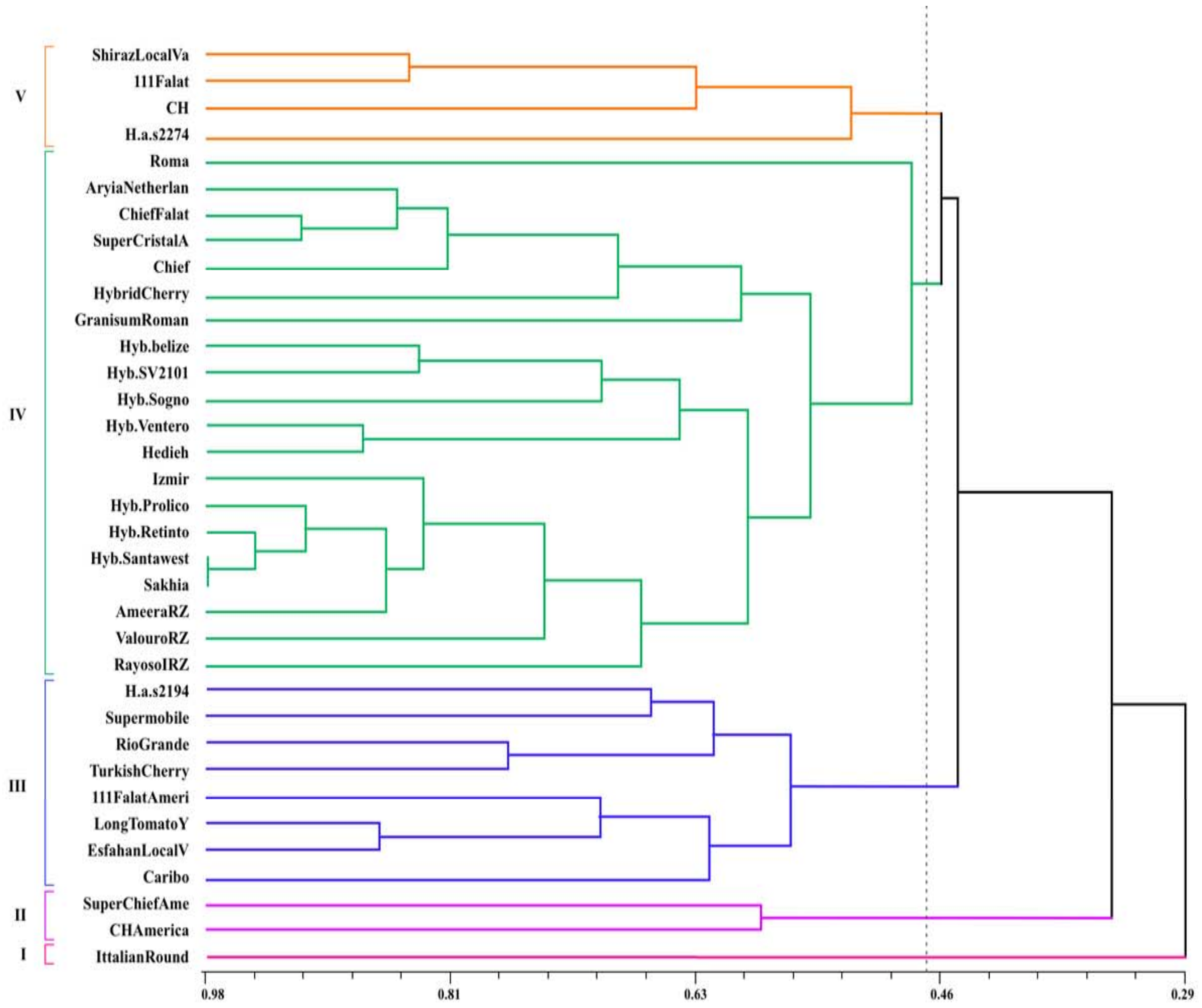

Supplemental Fig. 2. Clustering analysis based on 9 informative ISSR markers of 35 tomato genotypes using Jaccard's similarity coefficient and UPGMA algorithm.
