# Supplementary material for: Genomic markers analysis associated with resistance to Alternaria alternata (fr.) keissler—tomato pathotype, Solanum lycopersicum L
Source: Breed Sci. 2022 Aug 26;72(4):285–96. doi: 10.1270/jsbbs.22003 (PMC9868332; doi:10.1270/jsbbs.22003)
Supplement: Supplementary file 2 — Supplemental Tables [file 72_285_s2.pdf]

Alizadeh-Moghaddam et al./Breeding Science
doi: 10.1270/jsbbs.22003

Supplemental Table 1. The impact of tomato genotypes including genotype number, registered name, origin, properties, mean disease severity and reaction to Alternaria alternata

Code N.	[a]Registered name	[a]Status	[a]Origin/ Company	Disease (%) severity/Stages Means	[b]Reaction to A. alternata	Code N.	[a]Registered name	[a]Status	[a]Origin/ Company	severity/Stages Disease (%) Means	[b]Reaction to A. alternata
1	Shiraz local Variety	Local	IRAN / Danab	^{i}31.08	PS	19	Hybrid Cherry	Hybrid	Italy-Iran / Pakan Bazr	^{j}30.50	PS
2	H.a.s 2194	Hybrid	Iran / Pakan Bazr	^{o}27.33	PR	20	Romania Granisum	Hybrid	Romania / Pakan Bazr	^{lm}28.50	PS
3	Falat 111 American	Hybrid	USA / Gardesco	^{r}24.83	PR	21	Super Chief American	Hybrid	USA /Gardesco	^{ij}30.75	PS
4	Long Tomato Y	Hybrid	France / Griffaton	^{o}27.33	PR	22	CH American	Hybrid	USA / Us Agri. Seed	^{a}49.50	HS
5	Rio Grande	Hybrid	USA /Gardesco	^{v}14.83	RR	23	Hybrid Belize	Hybrid	USA / Seminis	^{k}29.50	PS
6	Turkish Cherry	Hybrid	France / Vilmoren	^{u}15.75	RR	24	Hybrid SV 1201	Hybrid	USA / Seminis	^{no}27.75	PR
7	H.a.s 2274	Hybrid	USA / Elit	^{t}22.50	PR	25	Hybrid Sogno	Hybrid	USA / Seminis	^{d}44.25	HS
8	Roma	Hybrid	USA / Hummert	^{q}25.50	PR	26	Hybrid Ventero	Hybrid	USA / Seminis	^{kl}29.00	PS
9	Esfahan local Variety	Local	Iran / Pakan Bazr	^{w}11.75	RR	27	Hedieh	Hybrid	France / Vilmoren	^{a}49.25	HS
10	Caribo	Hybrid	Netherlands / Golan Seed	^{h}31.75	PS	28	Izmir	Hybrid	Netherlands / Syngenta	^{b}47.25	HS
11	Supermobile	Hybrid	France / Griffaton	^{g}33.50	SS	29	Hybrid Prolico	Hybrid	USA / Seminis	^{kl}29.00	PS
12	Falat 111	Hybrid	USA / Elite	^{e}38.25	SS	30	Hybrid Retinto	Hybrid	USA / Seminis	^{a}49.25	HS
13	CH	Hybrid	Netherlands / Top Harryt	^{h}32.00	SS	31	Hybrid Santawest	Hybrid	USA / Seminis	^{f}34.50	SS
14	Italian Round	Hybrid	Italy / Ortosen	^{qr}25.25	PR	32	Sakhia (74-335 RZ)	Hybrid	Turkey / Rijk Zwaan	^{p}26.50	PR
15	Aryia Netherlands	Hybrid	Netherlands / Syngenta	^{mn}28.25	PS	33	Ameera RZ	Hybrid	Netherlands / Rex van	^{a}49.25	HS
16	Chief Falat	Hybrid	Iran / Falat	^{g}33.00	SS	34	Valouro RZ	Hybrid	Netherlands / Rex van	^{s}23.25	PR
17	Super Cristal American	Hybrid	USA /Genetics International	^{c}46.00	HS	35	Rayosol RZ	Hybrid	Netherlands / Rex van	^{q}25.75	PR
18	Chief	Hybrid	USA /Gardesco	^{lm}28.75	PS						

Note: [a]Reference from Moghaddam *et al*. 2020, [b]Reaction; RR (resistant), PR (partially resistant), PS (partially susceptible), SS (susceptible), HS (highly resistant).

Supplemental Table 2. The correlation analysis between 39 morphological traits and EB resistance level of 35 tomato genotypes

No.	Morphological Traits	Correlation	Resistance to *A. Alternata*	No.	Morphological Traits	Correlation	Resistance to *A. Alternata*	No.	Morphological Traits	Correlation	Resistance to *A. Alternata*
1	Plant size	Pearson Correlation	0.180*	14	Fruit pubescence	Pearson Correlation	-0.09	27	Seed colour	Pearson Correlation	-0.180**
		Sig. (2-tailed)	0.018			Sig. (2-tailed)	0.239			Sig. (2-tailed)	0.01
		N	175			N	175			N	175
2	Vine length	Pearson Correlation	0.576**	15	Predominant immature fruit shape	Pearson Correlation	-0.308**	28	Inflorescence type	Pearson Correlation	0.161*
		Sig. (2-tailed)	0			Sig. (2-tailed)	0			Sig. (2-tailed)	0.034
		N	175			N	175			N	175
3	Stem pubescence density	Pearson Correlation	0.308**	16	Exterior colour of mature fruit	Pearson Correlation	-0.344**	29	Corolla colour	Pearson Correlation	0.309**
		Sig. (2-tailed)	0			Sig. (2-tailed)	0			Sig. (2-tailed)	0
		N	175			N	175			N	175
4	Stem internode length	Pearson Correlation	0.181*	17	Mature Fruit size	Pearson Correlation	-0.338**	30	Corolla blossom type	Pearson Correlation	0.175*
		Sig. (2-tailed)	0.016			Sig. (2-tailed)	0			Sig. (2-tailed)	0.021
		N	175			N	175			N	175
5	Foliage density	Pearson Correlation	0.266**	18	Fruit width	Pearson Correlation	-0.058	31	Flower sterility type	Pearson Correlation	0.638**
		Sig. (2-tailed)	0			Sig. (2-tailed)	0.442			Sig. (2-tailed)	0
		N	175			N	175			N	175
6	Number of leaves under 1st inflorescence	Pearson Correlation	0.749**	19	Fruit length	Pearson Correlation	-0.360**	32	Petal length	Pearson Correlation	0.116
		Sig. (2-tailed)	0			Sig. (2-tailed)	0			Sig. (2-tailed)	0.127
		N	175			N	175			N	175
7	Leaf type	Pearson Correlation	-0.405**	20	Intensity of exterior colour	Pearson Correlation	-0.108	33	Petal width	Pearson Correlation	-0.174*
		Sig. (2-tailed)	0			Sig. (2-tailed)	0.16			Sig. (2-tailed)	0.022
		N	175			N	175			N	175
8	Degree of leaf dissection	Pearson Correlation	0.063	21	mature fruit shape	Pearson Correlation	-0.393**	34	Sepal length	Pearson Correlation	-0.298**
		Sig. (2-tailed)	0.408			Sig. (2-tailed)	0			Sig. (2-tailed)	0
		N	175			N	175			N	175
9	Anthocyanin colouration of Leaf Vein	Pearson Correlation	-0.033	22	Easiness of fruit to detach from the pedicel	Pearson Correlation	0.226**	35	Sepal width	Pearson Correlation	0.355**
		Sig. (2-tailed)	0.669			Sig. (2-tailed)	0.003			Sig. (2-tailed)	0
		N	175			N	175			N	175
10	Stem thickness	Pearson Correlation	0.306**	23	Fruit shoulder shape	Pearson Correlation	0.325**	36	Style position	Pearson Correlation	-0.264**
		Sig. (2-tailed)	0			Sig. (2-tailed)	0			Sig. (2-tailed)	0
		N	175			N	175			N	174
11	leaf blade width	Pearson Correlation	0.405**	24	Pedicel length	Pearson Correlation	-0.172*	37	Style shape	Pearson Correlation	-0.059
		Sig. (2-tailed)	0			Sig. (2-tailed)	0.023			Sig. (2-tailed)	0.442
		N	175			N	175			N	175
12	Leaf blade length	Pearson Correlation	0.211**	25	Thickness of pericarp	Pearson Correlation	-0.319**	38	Style hairiness	Pearson Correlation	0.266**
		Sig. (2-tailed)	0.005			Sig. (2-tailed)	0			Sig. (2-tailed)	0.001
		N	175			N	175			N	175
13	Exterior colour of immature fruit	Pearson Correlation	-0.115	26	Seed shape	Pearson Correlation	0.144*	39	Stamen length	Pearson Correlation	0.096
		Sig. (2-tailed)	0.129			Sig. (2-tailed)	0.036			Sig. (2-tailed)	0.205
		N	175			N	175			N	175

Note: **. Correlation is significant at the 0.01 level (2-tailed). *. Correlation is significant at the 0.05 level (1-tailed).

Supplemental Table 3. Summary of molecular data from 11 polymorphic ISSR markers among the 35 tomato genotypes: Primers, 5′→3′ Sequence, Temperature of Annealing, percent of Polymorphism, Band Frequency, Observed Number of Alleles (Na), Effective Number of Alleles (Ne), Polymorphic Information Content (PIC), Resolving Power Mean (Rp), Expected Heterozygosity (He), and Observed Heterozygosity Index (Ho).

Primer No.	Primers	*Sequence	Annealing Temperature	Polymorphic (%)	Band Frequency	Observed Number of Alleles (Na)	Effective Number of Alleles (Ne)	Polymorphic Information Content (PIC)	Resolving Power (Rp)	Expected Heterozygosity (H_e)	Observed Heterozygosity (H_o)
1	A7	T_{10}(AG)	54	100	0.472	1.857	1.498	0.351	0.754	0.318	0.285
2	UBC811	C_8(GA)	54	80	0.727	1.571	1.418	0.209	1.245	0.254	0.16
3	A12	CC_6(GA)	53	100	0.282	1.882	1.277	0.289	0.342	0.222	0.157
4	UBC855	YT_8(AC)	56	100	0.411	1.892	1.434	0.334	0.611	0.282	0.208
5	UBC818	G_8(CA)	54	100	0.336	1.842	1.304	0.291	0.468	0.227	0.171
6	UBC808	C_8(AG)	54	100	0.618	1.5	1.165	0.151	1.1	0.145	0.093
7	UBC840	YT_8(GA)	54	100	0.426	1.868	1.522	0.387	0.876	0.338	0.409
8	UBC880	$_3$(GGAGA)	53	100	0.394	1.929	1.452	0.348	0.497	0.299	0.203
9	INC3	YT_8(AC)	54	100	0.315	1.914	1.336	0.31	0.306	0.235	0.149
Mean				86.818	0.471	1.749	1.348	0.281	0.793	0.244	0.205

Note: * Y: T or C.
